# Thermal melanism explains macroevolutionary variation of dorsal pigmentation in Eurasian vipers

**DOI:** 10.1038/s41598-020-72871-1

**Published:** 2020-09-30

**Authors:** Fernando Martínez-Freiría, Ken S. Toyama, Inês Freitas, Antigoni Kaliontzopoulou

**Affiliations:** 1 CIBIO/InBIO, Research Center in Biodiversity and Genetic Resources of the University of Porto, Vairão, Portugal; 2 Department of Ecology and Evolutionary Biology, University of Toronto, Toronto, Canada

**Keywords:** Evolutionary ecology, Macroecology

## Abstract

Colouration may endorse thermoregulatory and antipredatory functions in snakes. The *thermal melanism hypothesis* predicts that dark-coloured individuals are ecologically favoured in cool climates. However, the loss of aposematic and cryptic colourations may imply high predation for melanistic snakes. Here, we used the monophyletic group of Eurasian vipers (subfamily Viperinae) to test whether an increase in the extent of dark area inside the characteristic zigzag dorsal pattern is associated to colder environments. We measured two colouration traits in zigzag-patterned individuals (number of dorsal marks and weighted pigmentation index) and used a phylogenetic comparative approach to explore macroevolutionary patterns of dorsal pigmentation and test whether its extent is associated to ecogeographic characteristics of lineages’ ranges. Phylogenetically-naïve and phylogenetically-informed analyses yielded a significant association between the degree of pigmentation of the zigzag pattern and environmental variables such as solar radiation, elevation and latitude. The degree of pigmentation of the zigzag pattern is highlighted as an adaptive trait that matches range attributes mirroring cold environments irrespective of the phylogeny. These results constitute the first large-scale evidence supporting the thermal melanism hypothesis in snakes, opening new avenues of inquiry for the mechanisms that shape the evolution of colour phenotypes.

## Introduction

Animals exhibit an enormous variability of colourations that has long fascinated and intrigued biologists^1^. Colouration is commonly involved in key aspects of animal ecology such as in predator–prey interactions where, for instance, background matching (i.e. crypsis) or bright/warning colourations (i.e. aposematism) have been extensively studied as strategies to avoid predator attacks^2^. Colouration is, therefore, considered an adaptive trait that is under strong selective pressures^3,4^.

In addition to other predation-related functions, colouration in reptiles might endorse a thermoregulatory function, as a consequence of their strong dependence on environmental temperature (i.e. due to ectothermy). In this respect, the *thermal melanism hypothesis* predicts an ecological advantage of dark colouration in cool climates, as dark-coloured individuals warm up faster and reach higher equilibrium temperatures than light-coloured individuals^5^. Studies developed with lizards, at distinct evolutionary levels and geographic scales, have provided support for this hypothesis (e.g.^6–8^). In snakes, intraspecific studies have indicated that melanistic individuals can exhibit increased growth rates, better body conditions and locomotor performances, longer activity periods, or higher female fertilities than non-melanistic ones^9–12^. Conversely, melanism or lack of dorsal pattern could increase detectability and thus, make dark snakes more vulnerable to predators^13–16^. This trade-off between predation and thermoregulation is likely behind the inability of the aforementioned studies to establish links between the occurrence of melanism and climatic factors, and therefore, to confirm the predictions of the thermal melanism hypothesis in snakes. Remarkably, such studies have only addressed variation in dorsal pigmentation within-species, despite the fact that the same ecological predictions apply across distinct taxa.

The relationship between colour phenotypes and predation has been recurrently explored in viperid snakes (Serpentes, Viperidae). Vipers frequently exhibit a zigzag dorsal pattern^17^ that endorses both aposematic and cryptic functions^15–19^. Melanistic vipers, however, do not exhibit the zigzag pattern. This lack of both cryptic and aposematic strategies, in conjunction with a high detectability due to the black colouration, leads to an increase of predation in melanistic vipers (e.g.^15^). As such, an alternative strategy to enhance thermoregulation in cold environments, while maintaining both aposematic and cryptic functions, would be by increasing the degree of dark pigmentation within the dorsal zigzag pattern. This can be achieved by augmenting the intensity of pigmented structures (i.e. increasing melanin and becoming darker); or by amplifying the extent of the dark area inside the dorsal design (i.e. increasing the number of scales with dark pigmentation).

Here we address the hypothesis that an increase in the extent of dark area inside the zigzag dorsal pattern might be associated to colder environments probably as a result of a balance between predation and thermoregulatory pressures, using the monophyletic group of Eurasian vipers (subfamily Viperinae) as a model system. Eurasian vipers are a highly diversified group of terrestrial venomous snakes (encompassing 4 genera and about 39 species) distributed across a wide variety of climates and environments (e.g. from arid Mediterranean to boreal, and from lowland to high elevation mountain ranges) in North Africa and Eurasia^20,21^. Although with distinct shape configurations (e.g. angular, rounded, stripped), all species exhibit the characteristic zigzag pattern, showing darker pigmentation inside it in relation to the background dorsal area^17^. We quantify dorsal pigmentation by measuring two colouration traits and then use a phylogenetic comparative approach to test whether the extent of dark dorsal pigmentation is associated to ecogeographic variation of species’ ranges. Specifically, we aim to answer the following questions: (1) Is variation in dorsal pigmentation evolutionarily constrained by phylogenetic history? (2) Does variation of dorsal pigmentation correlate with geographic and climatic descriptors of species distribution ranges? By answering these questions, we address the adaptive role of zigzag dorsal colouration in this group of vipers, testing its potential thermoregulatory function and seeking to identify its precise underlying environmental determinants.

## Results

**Figure 1:**
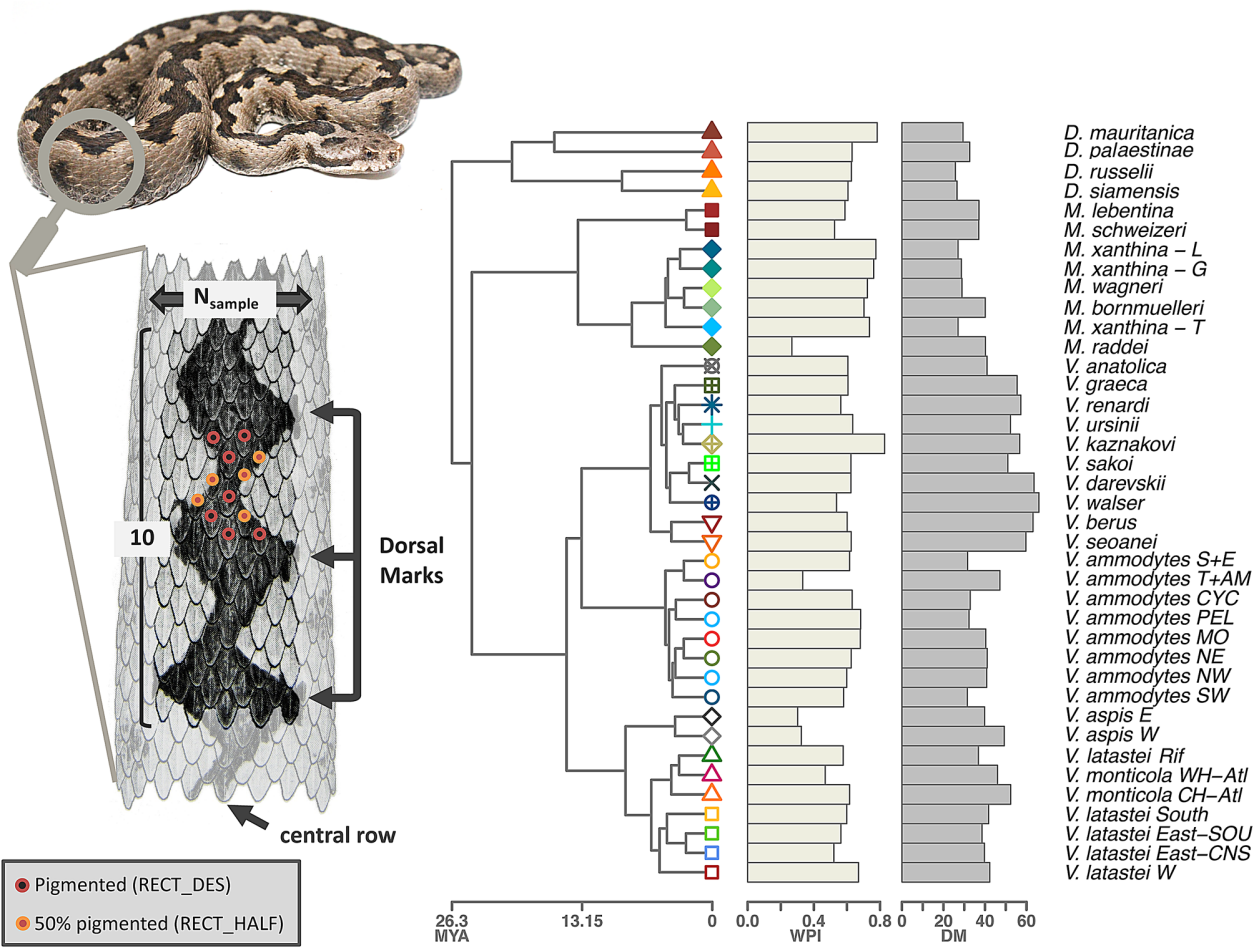
Schematic representation of the weighed pigmentation index (WPI) and the position of dorsal marks counted throughout the entire length of the body (DM), phylogenetic tree used for evolutionary analyses, and macroevolutionary variation in both traits across evolutionary units within Eurasian vipers. See Table S1 for evolutionary unit names.

**Figure 2:**
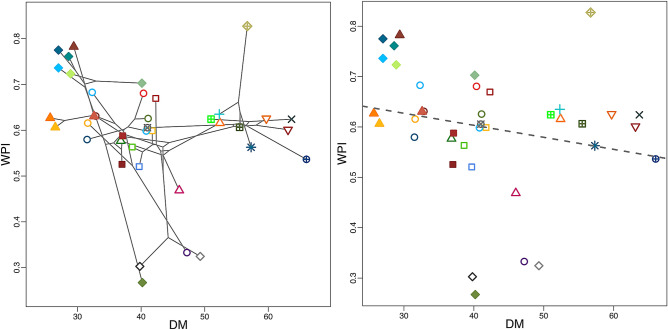
Phylomorphospace (left) and estimated PGLS regression describing the evolutionary association between the weighed pigmentation index (WPI) and the number of dorsal marks (DM) across evolutionary units within Eurasian vipers. See Fig. 1 for names of evolutionary unit symbols.

The macroevolutionary variation of dorsal pigmentation (Fig. 1) exhibited significant phylogenetic signal for both examined traits (DM: p = 0.001 ± 6.20 × 10^–5^; WPI: p = 0.016 ± 0.011), with a value close to that expected under a Brownian Motion model of evolution for DM (K = 0.956 ± 0.089), but with a much lower value for WPI (K = 0.390 ± 0.038). Phylogenetic generalized least-squares (PGLS) analyses showed a weak but significant, negative evolutionary covariation between both traits, where the degree of dorsal pigmentation (WPI) decreased as the number of dorsal marks (DM) increased (Table 1; Fig. 2).

**Table 1:** Summary ANOVA table for the evaluation of the evolutionary covariation between WPI and DM through PGLS.

	df	R^2^	F	Z	pval	pval < 0.05
DM	1	0.102 ± 0.011	4.195 ± 0.499	1.196 ± 0.06	0.048 ± 0.012	607
Residuals	37	0.898 ± 0.011				
Total	38					

Values given are means ± standard errors, as well as the number of significant p-values over 1000 phylogeny iterations (pval < 0.05).

**Figure 3:**
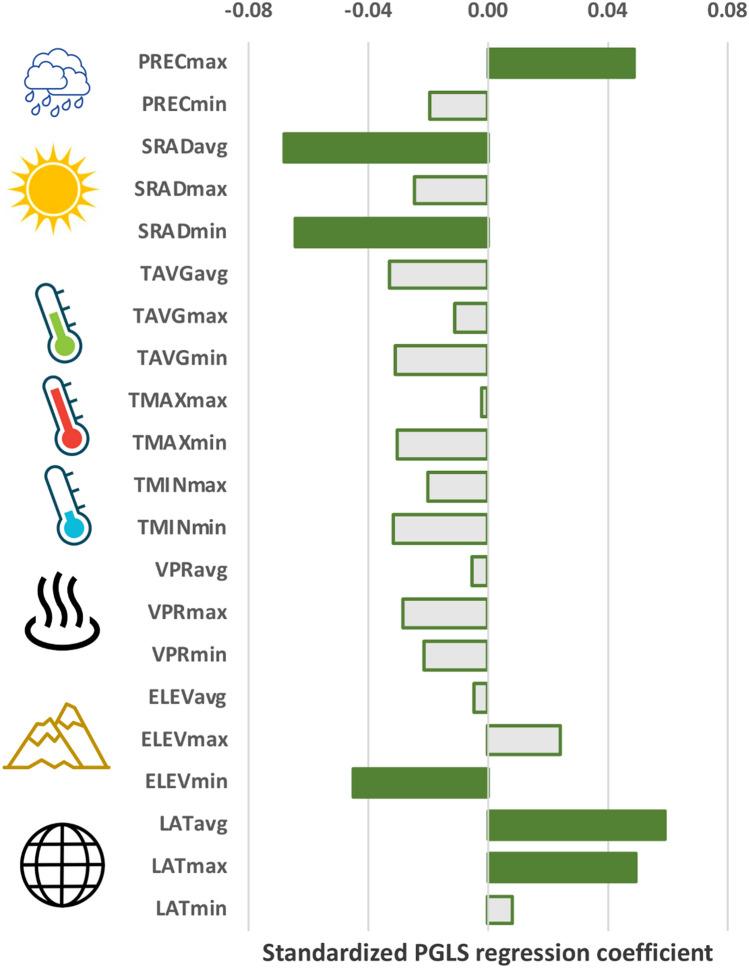
Estimated coefficients of PGLS models fit to examine the evolutionary covariation of the weighed pigmentation index (WPI) and range descriptors. Significant coefficients are marked in green. See main text for range descriptor codes.

Pairwise PGLS analyses between phenotypic traits and range variables identified a significant contribution of several environmental parameters in explaining macroevolutionary variation in the degree of dorsal pigmentation (WPI) (Supp. Table S2). Indeed, the WPI was positively associated to maximum precipitation, and to average and maximum latitude; and negatively associated to the mean and minimum value of solar radiation, and to minimum elevation (Fig. 3). By contrast, the number of dorsal marks (DM) was only associated to variation in the maximum value of the maximum temperature (Supp. Table S3).

Partial least-squares (PLS) analyses yielded significant vectors of association between pigmentation traits and range descriptors when ignoring phylogenetic effects. Examination of vector composition indicated that the multivariate association between both parameters was fully dominated by DM on the side of pigmentation traits (Table 2). This was also the case when phylogenetic effects were taken into account. Nevertheless, in this case, analyses combining both traits, or focusing on DM in isolation were non-significant. By contrast, both phylogenetically-naïve and phylogenetic PLS yielded a significant vector of association between WPI and environmental variation, where—in accordance with the results of pairwise PGLS—higher values of the WPI were associated with lower values of minimum solar radiation and elevation, and with higher values of maximum elevation and latitude (Table 2). By contrast, the association between DM and range descriptors was rendered non-significant when phylogenetic effects were taken into account.

**Table 2:** Vector loadings of PLS analyses between pigmentation traits and range descriptors.

	Phylogenetically naïve			Phylogenetically informed		
	Both traits	WPI	DM	Both traits	WPI	DM
WPI	− 3.41E−05	1		− 5.61E−04	1	
DM	1		1	0.628		1
PRECmax	− 0.053	0.212	− 0.053	− 0.021	0.253	− 0.029
PRECmin	0.109	− **0.306**	0.109	0.130	− 0.190	0.077
SRADavg	− 0.225	− 0.205	− 0.225	− 0.149	− 0.288	− 0.097
SRADmax	− 0.246	− 0.003	− 0.246	− 0.219	− 0.090	− **0.145**
SRADmin	− 0.162	− **0.346**	− 0.162	− 0.061	− **0.424**	− 0.038
TAVGavg	− 0.285	− 0.012	− 0.285	− 0.081	− 0.145	− 0.050
TAVGmax	− 0.328	0.136	− **0.328**	− 0.281	− 0.054	− **0.177**
TAVGmin	− 0.231	− 0.112	− 0.231	0.025	− 0.187	0.017
TMAXmax	− 0.326	0.160	− **0.326**	− 0.307	− 0.012	− **0.195**
TMAXmin	− 0.257	− 0.077	− 0.257	− 0.046	− 0.168	− 0.027
TMINmax	− 0.307	0.082	− **0.307**	− 0.212	− 0.117	− **0.131**
TMINmin	− 0.207	− 0.146	− 0.207	0.088	− 0.215	0.057
VPRavg	− 0.181	0.043	− 0.181	− 0.020	− 0.025	− 0.019
VPRmax	− 0.201	− 0.082	− 0.201	− 0.013	− 0.131	− 0.010
VPRmin	− 0.203	− 0.032	− 0.203	− 0.025	− 0.089	− 0.019
ELEVavg	0.145	− 0.211	0.145	0.085	− 0.047	0.051
ELEVmax	0.088	0.161	0.088	− 0.079	**0.315**	− 0.067
ELEVmin	0.200	− **0.611**	0.200	0.148	− **0.410**	0.109
LATavg	0.212	0.186	0.212	0.044	0.205	0.031
LATmax	0.177	**0.348**	0.177	− 0.085	**0.365**	− 0.052
LATmin	0.192	− 0.019	0.192	0.137	0.017	0.090
r	0.567	0.508	0.567	0.319 ± 0.039	0.557 ± 0.022	0.319 ± 0.039
p	0.001	0.008	0.001	0.502 ± 0.199	0.010 ± 0.006	0.502 ± 0.199

Variables with a relatively high contribution are highlighted in boldface.

*r* Pearson coefficient between PLS vectors, *p* corresponding p-value over 1000 iterations. For phylogenetically informed analyses means and standard errors of r and p over 1000 phylogenies are provided.

## Discussion

Our analysis of macroevolutionary variation in dorsal pigmentation in Eurasian vipers provides the first large-scale evidence supporting the thermal melanism hypothesis in snakes, and offers insights into how different phenotypic traits are constrained by evolutionary history and respond to geographic range attributes. The two traits used here to quantify dorsal pigmentation—the number of dorsal marks (DM) and a newly introduced weighed pigmentation index (WPI)—exhibit remarkably contrasting evolutionary trajectories, highlighting the relevance of considering both historical and ecological factors for explaining macroevolutionary phenotypic variation. Remarkably, our results pinpoint new perspectives on snake phenotypic evolution, emphasizing the potential for local adaptation through the modification of specific traits despite the existence of phylogenetic inertia in other, evolutionarily correlated, aspects of the phenotype.

Indeed, the analysis of the number of dorsal marks (DM) in Eurasian vipers performed here provides evidence for a high degree of phylogenetic inertia (sensu^22^), where macroevolutionary variation in this trait closely follows phylogenetic relatedness under a Brownian Motion model of evolution, as indicated by values of phylogenetic signal very close to one (K = 0.956 ± 0.089; see^23^). This reflects genus-level variation in DM across Eurasian vipers, where one may identify a clade that exhibits the highest values of DM (i.e. the *Pelias* clade, including *Vipera berus, V. seoanei,* and their allies of the *V. ursinii* group) and other monophyletic groups where the lowest values of DM occur (i.e. the genera *Daboia* and *Montivipera*) (Fig. 1). This confirms the long-standing notion that this trait is informative of evolutionary history in Eurasian vipers, as reflected by its common use for taxonomic purposes at both interspecific (e.g. in *Daboia/Macrovipera* species^24^; in the *V. ursinii* group^25^) and intraspecific levels (e.g. in *V. latastei-monticola* complex^19,26^; in *V. seoanei*^27^). While variation across lineages in DM can be linked to range descriptors through phylogenetically-naïve statistical procedures, such associations are rendered non-significant once shared evolutionary history is taken into consideration (Table 2). This highlights a strong phylogenetic inertia in this trait, where phenotype-environment links are rather outcomes of shared evolutionary history. Indeed, in the two extremes of variation for DM mentioned above, we observe clades that inhabit markedly different geographic areas, with *Daboia* and *Montivipera* inhabiting southern and mid-latitude areas within the entire clade of Eurasian vipers, while most members of the *Pelias* clade of the genus *Vipera* rather occupy mid-latitude or northern regions (Supp. Fig. S1). As such, the statistical association between DM and range descriptors seems to be driven by the combination of phylogenetic and spatial autocorrelation typical of groups that have diversified in distinct geographical regions.

By contrast, our analyses allowed us to establish a direct association between WPI and range attributes irrespective of phylogenetic relationships among evolutionary units (Table 2, Fig. 3, Table S2), which comply with the general predictions of the thermal melanism hypothesis. These results, combined to a low, significant value for phylogenetic signal in this trait (K = 0.390 ± 0.038) support the idea that the relative area occupied by pigmented scales within the zigzag dorsal design typical of Eurasian vipers has evolved adaptively as a response to range characteristics. The detailed examination of this association through PGLS and phylogenetically-informed PLS analyses provides new insights on the possible underlying mechanisms. First, and as similarly reported for heliothermic lizards (see^6^), temperature does not seem to be the main driver of macroevolutionary variation in dorsal pigmentation. Instead, solar radiation is highlighted as one of the most relevant factors, where lineages living in areas of lower solar radiation tend to be more pigmented (Fig. 3; Table 2). In terms of thermal balance, dark pigmentation implies low skin reflectance and thus, darker individuals should heat faster and reach higher equilibrium temperatures, by absorbing more solar radiation, than light-coloured (high skin reflectance) individuals^28^. Therefore, following the thermal melanism hypothesis, it is reasonable to predict that in environments with low solar radiation darker individuals have an ecological advantage over lighter ones and thus, natural selection would favour the occurrence of darker individuals. Furthermore, elevation and latitude are also retrieved through both pairwise PGLS and phylogenetic-PLS analyses as relevant range properties for explaining variation in WPI across lineages. This result complies with the general notion that “colder areas” (i.e. representing distribution ranges that occur in more northern latitudes and/or in higher altitudes) may trigger the adaptive evolution of increased dorsal pigmentation. Counter-intuitively we observed a negative association between WPI and minimum elevation, while we would expect lineages with higher WPI to occupy ranges with higher minimum elevation, reflecting their restriction to colder environments. This result is probably the result of the delimitation of distributional ranges, which is likely biased towards lowlands. Additionally, in most variables, PLS vector loadings are relatively low which could suggest that other factors not examined here (such as melanin-protection to UV radiation, which increases with latitude and altitude^29^) may be contributing to the underlying mechanisms. The tendency of lineages of more northern ranges and that inhabit areas of lower solar radiation to be more pigmented, may be further enhanced by modifications in the colour properties of the scales. Empirically, this seems like a feasible hypothesis, as groups that inhabit northern areas (e.g. members of the *Pelias* clade within *Vipera*) tend to have darker-coloured scales composing their zigzag design, as compared to those distributed more to the south (e.g. *Daboia* or members of the *V. latastei* and *V. ammodytes* complexes), which are more light-coloured. Such a hypothesis would need to be formally tested in light of the phylogeny of Eurasian vipers to further reinforce the conclusions of this study, through the direct quantification of scale colour characteristics in living specimens (e.g.^30,31^).

Such a complementary approach would also allow a more profound assessment of the precise genetic and developmental mechanisms that control the macroevolutionary patterns and signals identified here. Colouration in reptiles is a function of complex interactions between structural and pigmentary components^32^. Specific genes are known to regulate colouration (e.g. pro-opiomelanocortin gene is related to melanism), with polymorphism in these genes typically correlated to the occurrence of colour morphs (e.g. in *V. aspis*^33^). High estimates of heritability indicate a simple Mendelian pattern of inheritance for both colour and colour pattern in snakes^34–36^. Nevertheless, embryonic development also plays a role in variation of dorsal colouration (e.g. variation in thermal conditions can lead to abnormalities in the dorsal pattern shape in *V. aspis*^37^). Furthermore, in many snakes, the configuration of dorsal pattern is subject to variation across ontogeny (e.g. in sea kraits^38^), although such changes have never been reported in Eurasian vipers. Although it is reasonable to hypothesize that the number of dorsal marks and the degree of dorsal pigmentation might be linked both in terms of inheritance and development, our results suggest distinct levels of phylogenetic inertia and evolutionary flexibility for them in Eurasian vipers. Indeed, while DM is tightly linked to phylogenetic relatedness among lineages, sharply reflecting their shared evolutionary history, the WPI is rather established as an adaptive trait which responds to range-level ecogeographic parameters. This contrast suggests a decoupling of both traits, potentially at the genetic and/or developmental level, which needs to be further investigated for a more comprehensive understanding of the microevolutionary mechanisms that shape the interspecific variation observed. Importantly, phenotypic responses to ecogeographic variation appear to be mainly driven by a combination of latitude, altitude, solar radiation, and precipitation, supporting the idea that the compound of environmental conditions linked to the ecogeographic characteristics of species’ ranges, rather than temperature alone, may underlie colour adaptation to environmental conditions.

The *thermal melanism hypothesis* has repeatedly been summoned to explain the high frequency of dark-coloured individuals and its variation within and across populations of distinct snake species. The narrow scale of analysis (i.e. at intraspecific level) and the omission of other potential pressures affecting phenotypic variability (e.g. predation) could be behind the inability of distinct studies to find support for this hypothesis. By taking a phylogenetic comparative approach, we take such considerations to the macroevolutionary level, and address an alternative strategy of snakes to enhance thermoregulation in cold environments, while maintaining antipredatory functions of the dorsal colouration. Our work pinpoints the adaptive role of dorsal pattern pigmentation in Eurasian vipers, a trait that, irrespective of the phylogeny, varies in accordance to range attributes mirroring cold environments, providing for the first time evidence to support the thermal melanism hypothesis in snakes.

## Methods

### Data collection

Data on dorsal pigmentation were obtained from both living and museum specimens. In the case of living specimens, measurements were carried out in accordance with the current ethics and regulation on the use of animals for scientific research (EU Directive 2010/63/EU), with permits from the national or regional environmental authorities where the fieldwork was developed (e.g. for Portugal, Instituto da Conservação da Natureza e das Florestas, 558/2016/CAPT, 858/2018/CAPT, 14150/2019/DRNCN/DGEFF; for Spain, Gobierno de La Rioja-A/2017/021, A/2018/022, A/2019/028, Gobierno del Principado de Asturias-2019/003003, Junta de Castilla y León-EP/CyL/31/2017, EP/CyL/56/2018, EP/CyL/27/2019; for Morocco, Haut Commisariat aux Eaux and Forets-HCEFLCD/DLCDPN/DPRN/CFF N°19/2015, 35/2018). Museum authorities (directors and/or curators) provided permit for data collection from museum specimens (see Supplementary Material Table S1 for visited museum collections). When possible, we selected specimens in order to adequately cover the distribution range of each species. In total, we examined 1638 specimens, representing 39 lineages of Eurasian vipers (i.e. 97.5% of currently recognised species within the group; Table S1).

For each specimen, we quantified two morphological traits related to the degree of pigmentation (Fig. 1): first, we counted the total number of dorsal marks (DM, quantified as the number of angles of the dorsal pattern at its right side, excluding the head and the tail), which is a classic colouration trait in vipers (e.g.^19,21,27,39^); second, we calculated a weighed pigmentation index (WPI) that quantified the number of dark-pigmented vs. background light-coloured dorsal scales in a standardized area, by extending the protocol implemented by^19^. Specifically, we delimited a sampling rectangle in the central part of the dorsal area of each specimen, with a length of ten dorsal scales (across the length of the body) and a width (N_sample_) proportional to the total number of transversal rows of scales of each species (see Table S1). Note that, the width of this sampling rectangle was specifically selected to include the entire dorsal design in all sampled specimens, while at the same time avoiding the inclusion of the lateral parts of the body, and also accounting for the inter- and intraspecific variability observed in the number of dorsal rows in Eurasian vipers. Across this rectangle, we counted the total number of fully pigmented scales (RECT_DES) and the number of half-pigmented scales (RECT_HALF), out of the total number of scales included in the rectangle (10 × N_sample_; Fig. 1). Based on these values, we calculated the WPI as:

$${\text{WPI}} = \frac{{1 \times {\text{RECT}}\_{\text{DES}} + 0.5 \times {\text{RECT}}\_{\text{HALF}}}}{{10 \times {\text{N}}_{{{\text{sample}}}} }}$$

This index provides a measure of the degree of pigmentation across the dorsal area, weighed for the total number of dorsal scales present in each species. This index is thus particularly suitable when using specimens from museum collections in which preservation fluids (i.e. ethanol, formaldehyde) frequently alter colours, hampering the use of other methods to quantify colouration (e.g. analysis of digital photographs using pixel colour spectra^30^; quantitative point spectrophotometry^31^).

### Phylogenetic trees

In order to understand the relative role of phylogenetic relatedness in shaping dorsal pigmentation variation in Eurasian vipers, we first estimated the phylogenetic relationships within the group and delimited the evolutionary units for which subsequent analyses were based on.

For the phylogenetic reconstruction, we used the mitochondrial DNA dataset and the methodology applied in^21^. The dataset included seven mitochondrial gene fragments (CR—control region; COI—cytochrome *c* oxidase subunit I; cytb—cytochrome *b*; ND2—NADH dehydrogenase subunit 2; ND4—NADH dehydrogenase subunit 4; ND5—NADH dehydrogenase subunit 5; 16S—mitochondrial gene coding for 16S rRNA) and 93 evolutionary units representing most species/lineages within the group. Sequences were obtained from GenBank or generated by^21^ and selected according to their origin (concatenation was only performed when sequences originated from the same lineage or geographic location). Phylogenetic relationships and the time of divergence among species were inferred using Bayesian Inference (BI) as implemented in BEAST v 1.7.5^40^. The best-fitting model of molecular evolution and partition scheme were identified under the Bayesian Information Criterion (BIC; GTR + G + I for all genes combined in a single partition) using PartitionFinder 1.1.1^41^. A strict molecular clock and a Yule model were used as tree priors. Molecular dating relied on a single calibration point: the split of *Vipera*-*Daboia* and *Macrovipera*-*Montivipera* dated at 26 Mya (lognormal distribution: mean = 26, sd = 0.07)^21^. Three independent runs of 100 million generations were performed, sampling trees and parameter estimates every 10,000 generations with 10% of the trees discarded as burn-in. Convergence of the chains was confirmed using Tracer v1.6^42^. Trees obtained from multiple independent runs were then combined using LogCombiner v 1.7.5^40^ and summary trees were generated with TreeAnnotator v1.7.1^40^.

In order to exclude lineages that result from recent diversification events, the generated tree was cut at 2.5 Mya using the TreeSim R package^43^ allowing the delimitation of 40 evolutionary units, from which 39 were used in the following analyses (*Macrovipera razii* was excluded due to the lack of phenotypic data available; see Fig. 1). A new phylogeny was generated for these lineages, using the same priors and a single run of 100 million generations with sampling each 10,000 states and 10% of the trees discarded as burn-in. The quality of the run was verified using Tracer v1.6. To take phylogenetic uncertainty into account in subsequent phenotypic analyses, 1000 trees were then randomly sampled from the phylogeny posterior distribution.

### Distribution range data

For each of the 39 lineages, we obtained distribution range estimates by overlapping distribution polygons from the IUCN red list (https://www.iucnredlist.org/) and publications (e.g.^44–46^), and occurrences obtained from public databases (e.g. Global Biodiversity Information Facility, https://www.gbif.org/) and publications (e.g.^47,48^), together with recent phylogenetic studies (e.g.^49–51^) that further aided the delimitation of specific taxa (see Supp. Mat. Fig. S1 for range maps). Distribution ranges were estimated at 10 min of resolution (~ 20 km^2^) in the WGS 1984 datum.

From these distribution range maps, we measured range geographic position as described by average (LATavg), maximum (LATmax) and minimum (LATmin) latitude, and characterized the macro-ecological niche of lineages using topographic (elevation; from NASA’s Shuttle Radar Topography Mission, https://www.jpl.nasa.gov/srtm) and climatic variables (average, minimum and maximum temperature, precipitation, solar radiation and water vapour pressure; from the WorldClim database ver. 2, https://www.worldclim.org). To enhance accuracy in the calculation of geographic and climatic descriptors, we filtered out extreme values in variable distributions (i.e. 5% lower and upper quantiles) within delimited ranges (except in those lineages with less than 20 cells of range size). The downloaded climatic variables were composed of 12 layers each, one per month. For temperature-related variables, we first computed range average values for each lineage. After that, we calculated average (TAVGavg), maximum (TAVGmax) and minimum monthly temperatures (TAVGmin) for the average temperature observed across each lineage’s range; as well as maximum monthly temperatures (TMAXmax and TMINmax) and minimum monthly temperatures (TMAXmin and TMINmin) for maximum and minimum temperature across each lineage’s range. For water vapour pressure, we calculated mean values for individual lineage ranges and, from those, derived average monthly (VPRavg), maximum monthly (VPRmax), and minimum monthly (VPRmin) vapour pressure values. For precipitation and solar radiation, we added monthly layers to estimate total annual precipitation and solar radiation. Then, we computed average (SRADavg), maximum (SRADmax) and minimum (SRADmin) annual values of solar radiation, and maximum (PRECmax) and minimum (PRECmin) annual values of precipitation. We also extracted average (ELEVavg), maximum (ELEVmax) and minimum (ELEVmin) elevations across the range of each lineage. All variables were extracted at 10 min of resolution. All calculations were performed in ArcGis ver. 10.5^52^ and R ver. 3.4.4^53^, using the packages *raster* and *ncdf4*. Note that, although the calculated climatic variables are by definition numerically correlated with each other, they are all relevant to our hypotheses, as they represent average and extreme climatic conditions which may have contributed to local adaptation and lineage differentiation. By implementing adequate statistical procedures, not influenced by predictor variable collinearity (see below), we made sure to avoid statistical biases while maintaining all potentially relevant climatic range descriptors under consideration.

### Phylogenetic comparative analyses

To obtain species-level measures of morphological traits for phylogenetic comparative analyses, we first assigned individuals to each of the 39 lineages of Eurasian vipers by overlapping the coordinates of collection localities and distribution ranges. Then, we filtered out all specimens without an evident zig-zag pattern (i.e. melanistic, stripped or uniform individuals), which were markedly skewed only towards a few species (i.e. *V. berus, V. seoanei* and *V. kaznakovi*), finally retaining 1532 specimens for analyses (see Supp. Mat. Table S1 for number of specimens across lineages). From these, and in order to account for sampling bias, we retained the ten largest individuals (or those available, for lineages with n < 10), from which we calculated the mean value of DM and WPI corresponding to each tip of the phylogeny (Fig. 1).

Based on these species-level values, we first estimated Blomberg’s K^23^ to evaluate the strength of phylogenetic signal in each pigmentation trait, as implemented in the *phylosig* function of the *phytools* R-package^54^. In continuation, we described the evolutionary association between pigmentation traits through a PGLS regression of WPI on DM. To test whether environmental factors influenced the evolution of phenotypic variation, we performed pairwise PGLS regressions, in this case between each pigmentation trait and each of the range descriptors (i.e. geographic and climatic range variables). The significance of all PGLS models was evaluated using residual randomization procedures as implemented in the R-package *RRPP*^55,56^. Finally, to review the multivariate association between pigmentation and range characteristics, we used partial least squares analysis (PLS). To gain insights into the contribution of shared phylogenetic history in shaping multivariate association patterns, we performed these analyses both on raw species values, and taking phylogeny into account^57^. Furthermore, to also weigh the relative contribution of WPI and DM in driving the observed associations, we conducted these analyses combining both traits, but also for each trait in isolation. PLS analyses were implemented using the functions *two.b.pls* (for phylogenetically-naïve analyses) and *phylo.integration* (for phylogenetically-informed analyses), both from the *geomorph* R-package^58^.

All aforementioned analyses were repeated using the 1000 trees sampled from the phylogeny posterior distribution as explained above, to account for phylogenetic uncertainty and obtain the reported means and confidence intervals for all statistical descriptors and corresponding p-values. Manipulation of phylogenetic trees and phenotypic data were performed using the R-packages *ape*^59^ and *geiger*^60^. All analyses were performed using the R Language for Statistical Programming^53^.

## Supplementary information

Supplementary Information.

## Data Availability

The datasets generated and analysed during the current study are available from the corresponding author on reasonable request.
