# Supplementary material for: Thermal melanism explains macroevolutionary variation of dorsal pigmentation in Eurasian vipers
Source: Sci Rep. 2020 Sep 30;10:16122. doi: 10.1038/s41598-020-72871-1 (PMC7528074; doi:10.1038/s41598-020-72871-1)
Supplement: Supplementary file 1 — Supplementary Information. [file 41598_2020_72871_MOESM1_ESM.pdf]

**Thermal melanism explains macroevolutionary variation of dorsal pigmentation in Eurasian vipers.**

Fernando Martínez-Freiría[1*], Ken S. Toyama[2], Inês Freitas[1], Antigoni Kaliontzopoulou[1]

1. CIBIO/InBIO – Research Center in Biodiversity and Genetic Resources of the University of Porto, Vairão, Portugal; 2. Dep. Ecology and Evolutionary Biology, University of Toronto, Canada

* corresponding author, email: fmartinez-freiria@cibio.up.pt

**Supplementary Material**

## Supplementary Tables

**Table S1** – Lineage name (LINEAGE), number of specimens according to lineage (N), species/clades considered within each lineage (SPECIES), average (and range) of total dorsal rows (N rows), sampled dorsal rows (N sample) and source from where specimens were measured (SOURCE: museum collections visited / fieldwork performed by authors / personal collections of colleagues)

| LINEAGE | SPECIES | N | N rows | N sample | SOURCE |
|---|---|---|---|---|---|
| *D. mauritanica* | *Daboia mauritanica* | 71 | 26.6 (25-27) | 11 | collections: MNCN-M, MNHN-P, CEFE, NHM-L, NHM-V, UT, ZFMK / fieldwork |
| *D. palaestinae* | *Daboia palaestinae* | 23 | 25.1 (25-27) | 11 | collections: MNCN-M, MNHN-P, NHM-L, NHM-V |
| *D. russelii* | *Daboia russelii* | 51 | 28.7 (29-31) | 13 | collections: MNHN-P, NHM-L, NHM-V, ZFMK |
| *D. siamensis* | *Daboia siamensis* | 18 | 28.5 (27-31) | 13 | collections: NHM-L, ZFMK |
| *M. lebetina* | *Macrovipera lebetina* | 39 | 24.9 (23-25) | 11 | collections: MNHN-P, NHM-L, NHM-V, NM-P, ZFMK |
| *M. schweizeri* | *Macrovipera schweizeri* | 13 | 22.9 (21-23) | 9 | collections: NHM-L, NHM-V, NM-P, ZFMK |
| *M. bornmuelleri* | *Montivipera bornmuelleri* | 9 | 22.3 (21-23) | 9 | collections: NHM-L, NHM-V / personal collections: K. Mebert |
| *M. raddei* | *Montivipera latifii, Montivipera raddei* | 50 | 22.9 (21-23) | 9 | collections: MNHN-P, NHM-L, NHM-V, NM-P, ZFMK / personal collections: K. Mebert |
| *M. wagneri* | *Montivipera bulgardaghica, M. wagneri* | 36 | 23 (23) | 9 | collections: MNHN-P, NHM-V, NM-P, ZFMK / personal collections: K. Mebert |
| *M. xanthina* - G | *Montivipera xanthina*, Greek clade | 31 | 23 (23) | 9 | collections: NHM-L, NHM-V, ZFMK / personal collections: K. Mebert |
| *M. xanthina* - L | *Montivipera xanthina*, Lycian clade | 6 | 23 (23) | 9 | collections: NHM-V / personal collections: K. Mebert |
| *M. xanthina* - T | *Montivipera xanthina*, Taurus clade | 5 | 23 (23) | 9 | collections: NHM-V, ZFMK / personal collections: K. Mebert |
| *V. ammodytes* CYC | *Vipera ammodytes*, Cyclades clade | 8 | 21 (21) | 9 | collections: NHM-L, ZFMK |
| *V. ammodytes* MO | *Vipera ammodytes*, Montenegro clade | 14 | 21.6 (21-23) | 9 | collections: NHM-L, NHM-V, ZFMK |
| *V. ammodytes* NE | *Vipera ammodytes*, NE clade | 16 | 21 (21) | 9 | collections: NHM-L, NHM-V |
| *V. ammodytes* NW | *Vipera ammodytes*, NW clade | 77 | 21.4 (21-23) | 9 | collections: MNCN-M, MNHN-P, NHM-L, NHM-V, NM-P, ZFMK |
| *V. ammodytes* PEL | *Vipera ammodytes*, Peloponeso clade | 20 | 21 (21) | 9 | collections: MNHN-P, NHM-L, ZFMK / fieldwork |
| *V. ammodytes* S+E | *Vipera ammodytes*, South and and East clades | 50 | 21.04 (21-23) | 9 | collections: MNCN-M, MNHN-P, NHM-L, NHM-V, NM-P, ZFMK / fieldwork |
| *V. ammodytes* SW | *Vipera ammodytes*, SW clade | 10 | 21.2 (21-23) | 9 | collections: NHM-L, NHM-V |

| | | | | | |
|---|---|---|---|---|---|
| *V. ammodytes* T+AM | *Vipera ammodytes*, Turkish and Asian Minor clades | 14 | 21.1 (21-23) | 9 | collections: NHM-L, NHM-V, ZFMK |
| *V. anatolica* | *Vipera anatolica* | 29 | 19 | 7 | collections: MNHN-P / personal collections: K. Mebert |
| *V. aspis* E | *Vipera aspis*, East clade | 60 | 20.9 (19-21) | 9 | collections: MNHN-P, NHM-L, NHM-V, NM-P, ZFMK |
| *V. aspis* W | *Vipera aspis*, West clade | 110 | 21.02 (19-23) | 9 | collections: AR, EBD, IPE, MNC-B, MNCN-M, MNHN-P, CEFE, NHM-L, NHM-V, NM-P, UB, USAL, ZFMK |
| *V. berus* | *Vipera berus (barani* and *nikolskii* included) | 280 | 21 (19-23) | 9 | collections: MNCN-M, MNHN-P, NHM-L, NHM-V, NM-P, ZFMK |
| *V. darevskii* | *Vipera darevskii* | 20 | 20.9 (19-21) | 9 | collections: MNCN-M, NM-P / personal collections: K. Mebert |
| *V. graeca* | *Vipera graeca* | 28 | 19 (19) | 7 | collections: NHM-L, NHM-V, ZFMK / personal collections: E. Miszei |
| *V. kaznakovi* | *Vipera kaznakovi, V. dinniki, V. orlovi* | 46 | 21 (21-23) | 9 | collections: NHM-L, NHM-V, NM-P, ZFMK / personal collections: K. Mebert |
| *V. latastei* East-CNS | *Vipera latastei*, Iberia, Eastern-CNS clade | 31 | 21 (21) | 9 | collections: EBD, USAL, ZFMK / fieldwork |
| *V. latastei* East-SOU | *Vipera latastei*, Iberia, Eastern-SOU clade | 24 | 21 (21) | 9 | collections: EBD, MNCN-M / personal collections: X. Santos |
| *V. latastei* South | *Vipera latastei*, Southern clade | 14 | 21 (21) | 9 | collections: EBD, MNCN-M / fieldwork / personal collections: X. Santos, F. Jiménez Cazalla |
| *V. latastei* W | *Vipera latastei*, Iberia, Western clade | 60 | 20.9 (19-21) | 9 | collections: EBD, MHN-Li, MNCN-M, CEFE, NHM-V, USAL / fieldwork / personal collections: J.C. Brito |
| *V. monticola* CH-Atl | *Vipera latastei* (*monticola*), Central High Atlas | 17 | 19 (19) | 7 | collections: MNCN-M, NHM-V / fieldwork / personal collections: J.C. Brito |
| *V. latastei* RIF | *Vipera latastei*, Rif, Eastern Atlas and Algerian clade | 15 | 20.7 (19-23) | 9 | collections: MNCN-M, CEFE, NHM-L / fieldwork / personal collections: J.C. Brito |
| *V. monticola* WH-Atl | *Vipera latastei* (*monticola*), Western High Atlas | 2 | 19 (19) | 7 | fieldwork |
| *V. renardi* | *Vipera renardi, V. lotievi, V. altaica, V. eriwanensis* | 55 | 20.9 (19-21) | 9 | collections: MNHN, NHM-V, NM-P, ZFMK |
| *V. sakoi* | *Vipera sakoi* | 11 | 20.8 (19-21) | 9 | personal collections: K. Mebert |
| *V. seoanei* | *Vipera seoanei* | 165 | 21 (19 -23) | 9 | collections: AR, BIC, EBD, MNCN-M, MNHN-P, UO, UA / fieldwork / personal collections: J.C. Brito |
| *V. ursinii* | *Vipera ursinii* | 91 | 19.1 (19-21) | 7 | collections: NHM-L, NHM-V, NM-P, ZFMK |
| *V. walser* | *Vipera walser* | 19 | 20.8 (19-21) | 9 | personal collections: S. Ghielmi |

Acronyms for museum collections: AR (Sociedad de Ciencias Aranzadi, Donosti, Spain), BIC (Paisagem Protegida do Corno do Bico, Paredes de Coura, Portugal), EBD (Estación Biológica de Doñana – CSIC, Sevilla, Spain), IPE (Instituto Pirenaico de Ecología, Jaca, Spain), MCN-B (Museu de Ciències Naturals, Barcelona, Spain), MHN-Li (Museu Nacional de História Natural e da Ciência, Lisboa, Portugal), MNCN-M (Museo Nacional de Ciencias Naturales – CSIC, Madrid, Spain), MNHN-P (Muséum National d´Histoire Naturelle, Paris, France), CEFE (Centre d'Ecologie Fonctionnelle & Evolutive – CNRS, Montpellier, France), NHM-L (Natural History Museum, London, UK), NM-P (National Museum, Prague, Czeck Republic), UO (Universidad de Oviedo, Oviedo, Spain), UC (Universidade da Coruña, A Coruña, Spain), UB (Universitat de Barçelona, Barçelona, Spain), UT (Université Abdelmalek Essaadi, Tetouan, Morocco), USAL (Universidad de Salamanca, Salamanca, Spain), ZFMK (Zoological Research Museum Alexander Koenig, Bonn, Germany).

**Table S2** - Mean values of ANOVA statistics from pairwise PGLS models fit to examine the evolutionary covariation of WPI and range descriptors. Significant p-values (at a = 0.05) are highlighted in boldface. The estimated value for the regression coefficient (Coef) and the number of significant p-values over 1000 phylogeny iterations are also provided (only for significant effects).

| Range descriptors | $R^2$ | F | Z | pval | Coef | pval<0.05 |
|---|---|---|---|---|---|---|
| PRECmax | 0.126 | 5.35 | 1.277 | **0.039** | 0.049 | 837 |
| PRECmin | 0.038 | 1.473 | 0.701 | 0.249 | -0.019 | |
| SRADavg | 0.201 | 9.318 | 1.549 | **0.006** | -0.068 | 1000 |
| SRADmax | 0.024 | 0.925 | 0.42 | 0.387 | -0.024 | |
| SRADmin | 0.28 | 14.393 | 1.737 | **0.003** | -0.064 | 1000 |
| TAVGavg | 0.05 | 1.949 | 0.838 | 0.18 | -0.033 | |
| TAVGmax | 0.007 | 0.254 | -0.116 | 0.632 | -0.011 | |
| TAVGmin | 0.061 | 2.413 | 0.923 | 0.141 | -0.031 | |
| TMAXmax | 0.001 | 0.032 | -1.605 | 0.897 | -0.002 | |
| TMAXmin | 0.054 | 2.098 | 0.87 | 0.161 | -0.031 | |
| TMINmax | 0.025 | 0.97 | 0.507 | 0.349 | -0.02 | |
| TMINmin | 0.071 | 2.845 | 1.012 | 0.116 | -0.032 | |
| VPRavg | 0.003 | 0.113 | -0.987 | 0.794 | -0.005 | |
| VPRmax | 0.04 | 1.535 | 0.722 | 0.237 | -0.029 | |
| VPRmin | 0.021 | 0.793 | 0.396 | 0.403 | -0.021 | |
| ELEVavg | 0.004 | 0.151 | -0.812 | 0.765 | -0.005 | |
| ELEVmax | 0.079 | 3.218 | 1.062 | 0.093 | 0.024 | |
| ELEVmin | 0.189 | 8.623 | 1.471 | **0.005** | -0.045 | 1000 |
| LATavg | 0.124 | 5.227 | 1.275 | **0.025** | 0.059 | 1000 |
| LATmax | 0.185 | 8.394 | 1.461 | **0.009** | 0.049 | 1000 |
| LATmin | 0.002 | 0.080 | -1.008 | 0.820 | 0.008 | |

**Table S3** - Mean values of ANOVA statistics from pairwise PGLS models fit to examine the evolutionary covariation of DM and range descriptors. Significant p-values (at a = 0.05) are highlighted in boldface. The estimated value for the regression coefficient (Coef) and the number of significant p-values over 1000 phylogeny iterations are also provided (only for significant effects).

| Range descriptors | $R^2$ | F | Z | pval | Coef | pval<0.05 |
|---|---|---|---|---|---|---|
| PRECmax | 0.001 | 0.021 | -1.655 | 0.902 | -0.115 | |
| PRECmin | 0.009 | 0.339 | 0.049 | 0.579 | 0.811 | |
| SRADavg | 0.032 | 1.232 | 0.63 | 0.292 | -2.355 | |
| SRADmax | 0.071 | 2.825 | | 0.108 | -3.744 | |
| SRADmin | 0.005 | 0.191 | -0.269 | 0.669 | -0.733 | |
| TAVGavg | 0.012 | 0.451 | 0.18 | 0.509 | -1.388 | |
| TAVGmax | 0.096 | 3.937 | 1.154 | 0.057 | -3.828 | |
| TAVGmin | 0.001 | 0.022 | -1.747 | 0.908 | 0.065 | |
| TMAXmax | 0.116 | 4.84 | 1.309 | **0.039** | -4.268 | 858 |
| TMAXmin | 0.005 | 0.169 | -0.37 | 0.706 | -0.732 | |
| TMINmax | 0.049 | 1.891 | 0.853 | 0.18 | -2.475 | |
| TMINmin | 0.004 | 0.162 | -0.417 | 0.706 | 0.638 | |
| VPRavg | 0.001 | 0.044 | -1.268 | 0.862 | -0.328 | |
| VPRmax | 0.001 | 0.04 | -1.234 | 0.856 | -0.332 | |
| VPRmin | 0.002 | 0.071 | -0.833 | 0.797 | -0.516 | |
| ELEVavg | 0.006 | 0.222 | -0.188 | 0.64 | 0.668 | |
| ELEVmax | 0.002 | 0.07 | -1.173 | 0.839 | -0.226 | |
| ELEVmin | 0.012 | 0.439 | 0.151 | 0.533 | 0.958 | |
| LATavg | 0.004 | 0.159 | -0.366 | 0.709 | 0.927 | |
| LATmax | 0.004 | 0.147 | -0.444 | 0.744 | -0.597 | |
| LATmin | 0.049 | 1.925 | 0.83 | 0.178 | 4.226 | |

**Supplementary Figure**

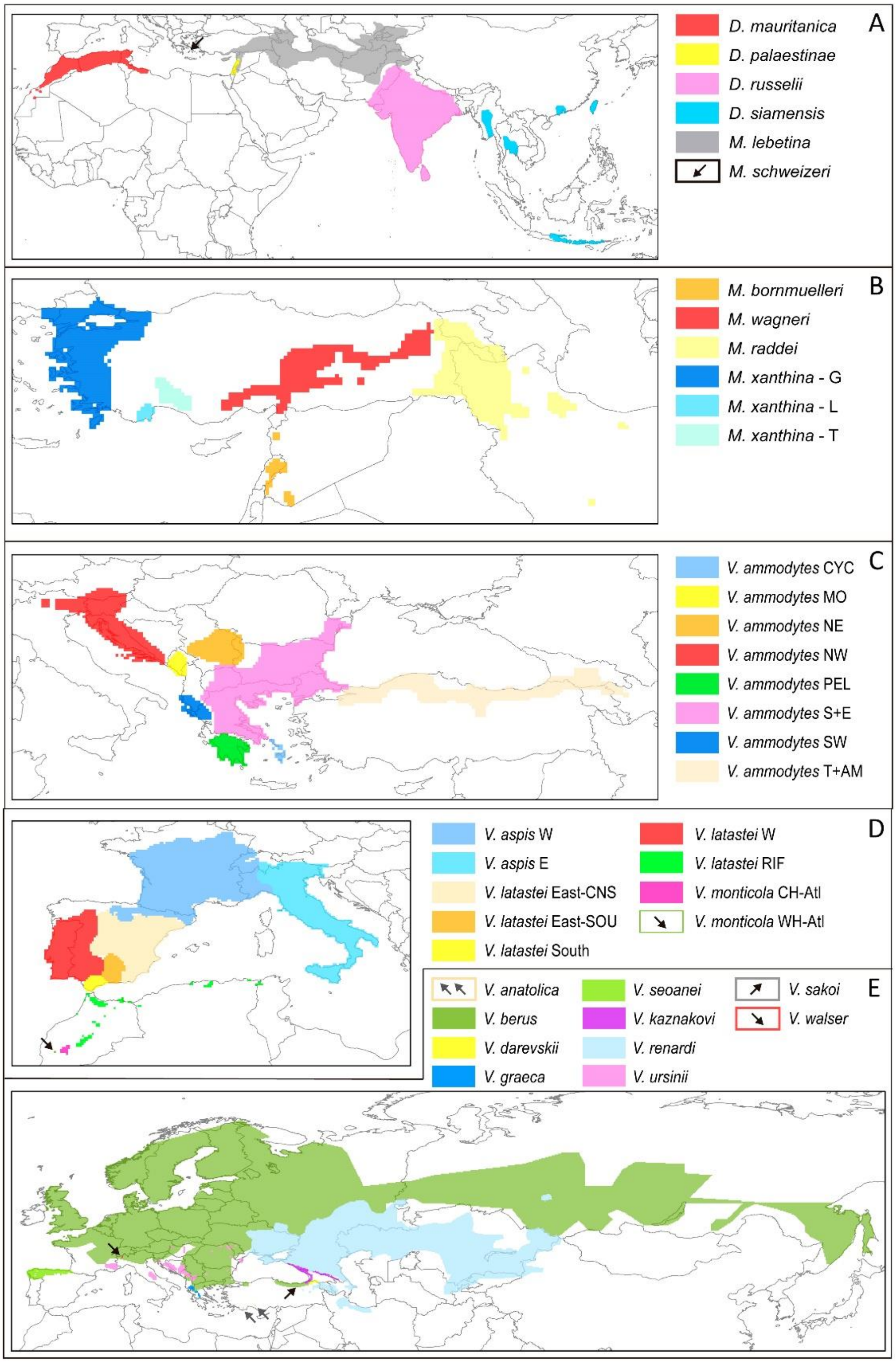


**Figure S1** – Distribution ranges for the 39 lineages of Eurasian vipers considered in this study, grouped accordingly to genera (A) *Daboia* and *Macrovipera*, (B) *Montivipera*, (C) *Vipera*, clade *Vipera* 2, (D) *Vipera*, clade *Vipera* 1, and (E) *Vipera*, clade *Pelias*. See Table S1 for lineages names.
